# Plasma amino acid profiles in healthy East Asian subpopulations living in Japan

**DOI:** 10.1002/ajhb.22787

**Published:** 2015-09-26

**Authors:** Hidehiro Nakamura, Natsumi Nishikata, Nobuhiro Kawai, Akira Imaizumi, Hiroshi Miyano, Maiko Mori, Hiroshi Yamamoto, Yasushi Noguchi

**Affiliations:** ^1^ Institute for Innovation, Ajinomoto Co., Inc KawasakiJapan; ^2^ Department of R&D PlanningAjinomoto Co., IncTokyoJapan

## Abstract

**Objectives:**

Profiles of plasma free amino acids (PFAAs) have been utilized as biomarkers to detect various diseases. However, few studies have investigated whether ethnicity or specific subpopulations within East Asia influence PFAA concentrations.

**Methods:**

A total of 95 healthy volunteers living in Japan, including 31 Japanese individuals, 36 Korean individuals and 28 Chinese individuals, were enrolled. Participants’ PFAA levels were measured by high‐performance liquid chromatography mass spectrometry, and the effects of factors such as sex, age, body mass index (BMI) and subpopulation on PFAA profiles were analyzed.

**Results:**

With the exception of glutamine and α‐aminobutyric acid, there were no significant differences among the three examined subpopulations with respect to either the means or the distributions of PFAA concentrations. A multiple regression analysis revealed that most of the PFAA concentrations were significantly related to sex. Ornithine concentrations, glutamate concentrations, and glutamine and α‐aminobutyric acid concentrations were significantly associated with age, BMI, and Chinese subpopulation, respectively.

**Conclusion:**

The study results indicate that the contributions of subpopulation within East Asia to PFAA profiles are small, particularly relative to the contributions provided by sex. Am. J. Hum. Biol. 28:236–239, 2016. © 2015 The Authors American Journal of Human Biology Published by Wiley Periodicals, Inc.

Amino acids (AAs) play important roles as both basic metabolites and physiological regulators. Although AA metabolism is strictly regulated in healthy individuals, reports have indicated that certain diseases influence plasma free amino acid (PFAA) profiles (Holm et al., 1999; Hong et al., 1998; Miyagi et al., 2011; Felig et al., 1970). Based on these PFAA characteristics, “AminoIndex Technology,” which can evaluate specific health conditions and disease possibilities by analyzing PFAA status, has recently been described (Miyagi et al., 2011; Okamoto 2011).

PFAA homeostasis is primarily maintained by the balance among the endogenous synthesis of nonessential AAs, the degradation of AAs, and the synthesis and breakdown of proteins. Because these reactions are performed by various enzymes, PFAA profiles may be influenced by genetic variations in enzymes involved in AA metabolism. In fact, a prior genome‐wide association study (GWAS) of the human metabolome indicated that certain AAs are associated with genetic loci encoding either AA transporters or enzymes involved in serine (Ser) biosynthesis (Rhee et al., 2013).

It has been reported that the frequency of single nucleotide polymorphisms in certain enzymes involved in drug metabolism is associated with ethnicity (Man et al., 2010). Genetic variations among three major East Asian subpopulations (Japanese, Korean and Chinese) have also been reported with respect to enzymes, including the CYP19*3 variant (Man et al., 2010), although variations are much smaller than between Europeans and Africans. However, there have been no reports regarding the genetic heterogeneity of enzymes involved in AA metabolism, and few studies have described the relationship between PFAA profiles and either ethnicity or subpopulations.

In this study, we compared the PFAA concentrations of healthy Japanese, Korean, and Chinese individuals living in Japan and investigated how subpopulation, as well as age, sex, and body mass index (BMI), affected PFAA profiles.

## MATERIALS AND METHODS

### Ethics statement

This study was conducted in accordance with the Declaration of Helsinki, and experimental protocols were approved by the ethics committee of Ajinomoto Co., Inc. All subjects provided written informed consent for their inclusion before participating in the study. All data were analyzed anonymously throughout the study.

### Enrollment

A total of 95 healthy volunteers, including 31 Japanese individuals, 36 Korean individuals and 28 Chinese individuals (86 percent of whom were Han Chinese) were recruited for this study. Each participant's subpopulation was determined using a questionnaire that verified the Japanese, Korean or Chinese backgrounds of both of the participant's parents. All subjects had been living in Japan for at least one year. Information about diet was based on a 3‐month recall interview. The frequency of food intake from their home countries (e.g., Korean food for Korean and Chinese food for Chinese) was investigated by choosing from “daily,” 4–6 times a week, “1–3 times a week,” “2–3 times month,” once a month or less often,″ and “never.” Fifty‐eight percent of Korean and 50% of Chinese consume their local food more than 4–6 times a week. The exclusion criteria of taking medication, pregnancy, mental disorders and cancer were applied at the beginning of the study.

### Quantification of plasma AA concentrations

Blood samples (5 ml) were collected from forearm veins in the morning following overnight fasting into tubes containing ethylenediaminetetraacetic acid. These samples were immediately placed on ice. Plasma was prepared via centrifugation at 2,010 g and 4°C for 15 min and stored at −80°C until needed for analysis. Subsequently, plasma samples were deproteinized with acetonitrile at a final concentration of 80%. The samples were then subjected to precolumn derivatization followed by high‐performance liquid chromatography (HPLC)‐electrospray ionization (ESI)‐mass spectrometry (MS) for AA quantification, which was performed as previously described (Shimbo et al., 2009a; Shimbo et al., 2009b; Yoshida et al., 2015). The following 21 AAs were quantified: alanine (Ala), alpha‐aminobutyric acid (α‐ABA), arginine (Arg), asparagine (Asn), citrulline (Cit), glutamate (Glu), glutamine (Gln), glycine (Gly), histidine (His), isoleucine (Ile), leucine (Leu), lysine (Lys), methionine (Met), ornithine (Orn), phenylalanine (Phe), proline (Pro), serine (Ser), threonine (Thr), tryptophan (Trp), tyrosine (Tyr), and valine (Val).

### Statistical analysis

Study data are presented as means ± standard deviation (SD). Statistical and multivariate analyses were performed using the JMP 9.0.0 program (SAS Institute Inc., NC). To assess differences among the three subpopulations, Tukey's test was performed following a one‐way analysis of variance (ANOVA). Levene's test was performed to assess the homogeneity of variances. Multiple linear regression (MLR) was used to evaluate the contributions of background factors (sex, age, BMI or subpopulation) to PFAA concentrations. *P* < 0.05 was established as the level of significance following Bonferroni multiple comparison tests.

## RESULTS

Table 1 presents the characteristics of the Japanese, Korean, and Chinese subjects enrolled in this study. The sex ratios of subjects in these three subpopulations did not differ. The mean age of the Korean participants was significantly lower than the mean age of the Japanese and Chinese participants. The mean BMI of the Chinese subjects was significantly higher than the mean BMI of the Japanese subjects.

**Table 1:** Plasma AA concentrations in three major East Asian subpopulations

	Japanese	Korean	Chinese	*P‐*value by ANOVA	*P‐*value by Levene's test
*N* (Male/Female)	31 (16/15)	36 (19/17)	28 (13/15)	NS	
Age (years)	46.5 ± 7.4^a^	37.2 ± 6.7^b^	45.1 ± 11.0^a^	<0.0001	0.0177
BMI (kg·m^−2^)	21.0 ± 1.9^b^	21.5 ± 2.4^a,b^	22.7 ± 2.7^a^	0.0236	0.0930
*Essential amino acids (μmol./L)*					
Val	213.6 ± 59.5 (134.3–368.1)	207.4 ± 40.4 (118.3–312.0)	224.7 ± 38.5 (156.4–282.5)	0.3419	0.0711
Ile	61.1 ± 19.4 (38.0–115.3)	61.7 ± 14.9 (33.6–104.2)	63.6 ± 15.2 (42.8–96.7)	0.8355	0.3469
Leu	118.6 ± 34.6 (70.1–206.0)	116.9 ± 27.8 (67.7–169.3)	127.2 ± 24.6 (85.2–177.1)	0.3510	0.4097
His	85.8 ± 10.6 (65.5–106.0)	83.2 ± 9.2 (63.9–99.2)	82.4 ± 9.2 (68.1–98.0)	0.3669	0.8350
Phe	57.5 ± 10.1 (45.5–89.5)	55.4 ± 9.6 (40.0–78.2)	59.0 ± 9.2 (46.3–88.8)	0.3237	0.6421
Trp	54.9 ± 11.8 (36.3–82.1)	56.8 ± 9.8 (36.1–83.5)	55.3 ± 9.6 (34.0–74.2)	0.7390	0.4979
Met	26.8 ± 5.8 (18.5–44.5)	27.7 ± 5.9 (18.5–40.6)	25.0 ± 6.5 (13.6–47.3)	0.1977	0.8616
Thr	133.7 ± 33.1 (81.3–235.5)	138.0 ± 29.4 (74.0–238.6)	119.2 ± 32.4 (62.2–192.2)	0.0558	0.5712
Lys	173.1 ± 31.4 (113.0–255.2)	181.9 ± 28.4 (131.8–249.7)	181.2 ± 38.1 (102.6–276.7)	0.4916	0.6991
*Nonessential amino acids (μmol./L)*					
Ser	113.3 ± 23.8 (62.1–179.1)	124.3 ± 20.8 (82.4–166.5)	116.2 ± 22.8 (80.3–161.1)	0.1198	0.6604
Gly	246.4 ± 58.9 (95.3–397.7)	240.1 ± 53.4 (119.1–381.0)	229.0 ± 40.4 (166.1–326.0)	0.4311	0.3460
Ala	384.6 ± 97.1 (250.1–595.6)	370.7 ± 94.0 (241.7–671.3)	372.1 ± 108.9 (196.2–681.5)	0.8292	0.7391
Tyr	59.8 ± 16.6 (38.3–116.9)	59.4 ± 13.6 (31.6–90.9)	62.5 ± 13.4 (40.5–107.2)	0.6642	0.7540
Glu	26.3 ± 12.0 (10.1–50.3)	24.1 ± 11.9 (8.3–64.9)	32.7 ± 20.1 (11.7–86.7)	0.0669	0.0351
Pro	146.2 ± 48.3 (67.1–271.2)	159.8 ± 49.7 (97.2–272.7)	150.5 ± 60.1 (73.2–348.2)	0.5573	0.6801
Gln	617.9 ± 65.1^a^ (474.8–743.8)	578.2 ± 62.9^a,b^ (402.4–706.4)	554.0 ± 70.7^b^ (437.7–700.0)	0.0013*	0.6794
Cit	31.1 ± 6.2 (17.5–41.7)	29.6 ± 7.0 (19.6–46.5)	30.2 ± 7.3 (16.5–42.9)	0.6699	0.5085
Arg	96.1 ± 20.6 (53.6–138.6)	101.0 ± 16.8 (69.5–135.8)	101.9 ± 25.9 (59.6–151.2)	0.5068	0.0103
Orn	47.9 ± 15.2 (17.8–83.3)	48.6 ± 10.6 (26.6–79.7)	50.0 ± 14.9 (24.2–83.5)	0.8256	0.1480
Asn	48.8 ± 5.6 (38.0–65.1)	48.2 ± 7.8 (34.2–71.5)	48.6 ± 9.7 (33.9–71.8)	0.9534	0.0591
α‐ABA	14.5 ± 3.6^b^ (9.4–22.9)	17.6 ± 5.4^a,b^ (10.1–35.0)	20.4 ± 6.5^a^ (9.0–36.0)	0.0003*	0.0355

Data are presented as means ± SD (range). The chi‐squared test was used to test for differences in sex distributions. Levene's test was performed to assess the homogeneity of variances. Significant differences among the three groups were determined by one‐way ANOVA followed by Tukey's test and are presented in bold. Different characters indicate significant differences (*P*<0.002). *A significance level of *P* < 0.002 was established (a Bonferroni‐corrected threshold).

The concentrations of 21 PFAAs in the examined subpopulations were compared (Table 1). Gln and α‐ABA concentrations were significantly lower and higher, respectively, in the Chinese subpopulation than in the Japanese subpopulation. No significant differences among subpopulations were observed with respect to the concentrations of the remaining 19 PFAAs. Additionally, there were no significant differences among the three subpopulations with respect to the variances in PFAA concentrations.

To investigate the contributions of sex, age, BMI, and subpopulation to PFAA concentrations, MLR analysis was applied (Table 2). The results of this analysis indicated that most of the PFAA concentrations were strongly related to sex. Age, BMI, and Chinese subpopulation significantly affected Orn, Glu, and Gln and α‐ABA concentrations, respectively. The Korean subpopulation factor had no effect on PFAA profiles.

**Table 2:** The influences of sex, age, BMI, and subpopulation on PFAA concentrations

	Factors				
	Sex	Age	BMI	Korean	Chinese
*Essential amino acids*					
Val	<0.0001*	0.9864	0.2051	0.4748	0.4115
Ile	<0.0001*	0.8451	0.1848	0.9490	0.6007
Leu	<0.0001*	0.4462	0.0444	0.4106	0.3622
His	0.0109	0.8981	0.3976	0.2475	0.1454
Phe	<0.0001*	0.3371	0.2166	0.5322	0.6071
Trp	<0.0001*	0.7086	0.8186	0.5838	0.7506
Met	<0.0001*	0.7231	0.1807	0.5383	0.1467
Thr	0.2730	0.4616	0.6383	0.8966	0.0757
Lys	0.0062	0.2230	0.0729	0.1564	0.5267
*Nonessential amino acids*					
Ser	0.4935	0.9850	0.3656	0.0704	0.4461
Gly	0.1227	0.3463	0.0289	0.8963	0.6650
Ala	0.0153	0.0772	0.1638	0.9306	0.4906
Tyr	0.0011*	0.3109	0.0214	0.9331	0.7926
Glu	0.0007*	0.5952	0.0004*	0.4555	0.3265
Pro	0.0003*	0.1851	0.5943	0.1309	0.6889
Gln	0.0231	0.0293	0.7393	0.1703	0.0006*
Cit	0.0050	0.0384	0.3357	0.8963	0.9601
Arg	0.0305	0.4427	0.1468	0.2804	0.4517
Orn	<0.0001*	<0.0001*	0.5965	0.0465	0.3004
Asn	0.0515	0.4589	0.4726	0.9258	0.7981
α‐ABA	0.1720	0.6870	0.0942	0.0335	0.0004*

Multiple linear regression was applied to estimate the effects of sex, age, BMI and subpopulation, and the resulting *P* values are presented (*a significance level of *P* < 0.002; Bonferroni‐corrected threshold). Significant differences are presented in bold.

## DISCUSSION

In the current study, there were no significant differences among subpopulations with respect to the distributions and mean levels of all examined PFAAs except for Gln and α‐ABA. Chinese subpopulation significantly influenced plasma concentrations of Gln and α‐ABA, even after the effects of background characteristics such as sex, age and BMI were eliminated. However, the concentrations of these AAs in most of the Chinese subjects were within reference intervals for Japanese subjects (431.0–691.6 μM for Gln and 11.2–31.6 μM for α‐ABA) (Yamamoto et al., 2015), suggesting that the PFAA profiles of Korean and Chinese individuals living in Japan do not greatly differ from those of Japanese individuals. Certain prior studies have examined the influence of ethnicity on PFAA profiles. Lawton et al. (2008) measured plasma metabolites in healthy Europeans, African‐Americans and Hispanics; their findings demonstrated that among these populations, ethnicity exerts only limited effects on concentrations of PFAAs. Another group measured PFAA concentrations in Greenlanders and Danes and reported that residence exerted the strongest influence on PFAA concentrations; ethnicity and diet had little to no effect on these concentrations, although ethnicity was weakly related to the plasma concentrations of Glu, His, Cit, and Arg (Pedersen et al., 2006). These data are consistent with our results, which indicate that differences among East Asian subpopulations do not provide large contributions to determining PFAA concentrations in healthy subjects. Additional studies are needed to clarify the effects of residence and dietary habits on PFAA profiles (e.g., Korean and Chinese subjects living in Japan for less than one year).

MLR analysis demonstrated that PFAA profiles, particularly with respect to essential AAs, were more strongly related to sex than to age, BMI or subpopulation. Our previous study also found that sex is a more influential factor for PFAA profiles than age or BMI (Yamamoto et al., 2015). In addition, reports have indicated that PFAA levels are strongly correlated with visceral fat area and insulin‐related parameters but not with subcutaneous fat area (Nakamura et al., 2014; Yamakado et al., 2012). Furthermore, the ingestion of a low‐protein diet leads to decreased PFAA levels (Fujita et al., 1978). These results suggest that PFAA levels may be more strongly influenced by sex, physiological conditions or dietary habits than by genetic factors.

In conclusion, we have demonstrated that there is little to no difference among the PFAA profiles of Japanese, Korean and Chinese individuals living in Japan, although this is a preliminary study and needs confirmation with larger numbers of subjects. These results suggest that it may be feasible to clinically utilize PFAA profiles for the detection of diseases such as cancer or metabolic syndrome, independent of differences among the three East Asian subpopulations. Further investigations are needed to confirm whether PFAA profile changes associated with these diseases and physiological conditions are similar among these subpopulations.

## References

[ajhb22787-bib-0001] Felig P , Marliss E , Ohman JL , Cahill CF Jr. 1970 Plasma amino acid levels in diabetic ketoacidosis. Diabetes 19(10):727–728. 4990780 10.2337/diab.19.10.727

[ajhb22787-bib-0002] Fujita Y , Yoshimura Y , Inoue G. 1978 Effect of low‐protein diets on free amino acids in plasma of young men: effect of protein quality with maintenance or excess energy intake. J Nutr Sci Vitaminol 24(3):297–309. 99500 10.3177/jnsv.24.297

[ajhb22787-bib-0003] Holm E , Sedlaczek O , Grips E. 1999 Amino acid metabolism in liver disease. Curr Opin Clin Nutr Metab Care 2(1):47–53. 10453330 10.1097/00075197-199901000-00009

[ajhb22787-bib-0004] Hong SY , Yang DH , Chang SK. 1998 The relationship between plasma homocysteine and amino acid concentrations in patients with end‐stage renal disease. J Ren Nutr 8(1):34–39. 9724828 10.1016/s1051-2276(98)90035-8

[ajhb22787-bib-0005] Lawton KA , Berger A , Mitchell M , Milgram KE , Evans AM , Guo L , Hanson RW , Kalhan SC , Ryals JA , Milburn MV. 2008 Analysis of the adult human plasma metabolome. Pharmacogenomics 9(4):383–397. 18384253 10.2217/14622416.9.4.383

[ajhb22787-bib-0006] Man M , Farmen M , Dumaual C , Teng CH , Moser B , Irie S , Noh GJ , Njau R , Close S , Wise S , Hockett R . 2010 Genetic variation in metabolizing enzyme and transporter genes: comprehensive assessment in 3 major East Asian subpopulations with comparison to Caucasians and Africans. J Clin Pharmacol 50(8):929–940. 20173083 10.1177/0091270009355161

[ajhb22787-bib-0007] Miyagi Y , Higashiyama M , Gochi A , Akaike M , Ishikawa T , Miura T , Saruki N , Bando E , Kimura H , Imamura F , Moriyama M , Ikeda I , Chiba A , Oshita F , Imaizumi A , Yamamoto H , Miyano H , Horimoto K , Tochikubo O , Mitsushima T , Yamakado M , Okamoto N . 2011 Plasma free amino acid profiling of five types of cancer patients and its application for early detection. PloS one 6(9):e24143. 21915291 10.1371/journal.pone.0024143 PMC3168486

[ajhb22787-bib-0008] Nakamura H , Jinzu H , Nagao K , Noguchi Y , Shimba N , Miyano H , Watanabe T , Iseki K. 2014 Plasma amino acid profiles are associated with insulin, C‐peptide and adiponectin levels in type 2 diabetic patients. Nutr Diabetes 4:e133. 25177913 10.1038/nutd.2014.32 PMC4183973

[ajhb22787-bib-0009] Okamoto N. 2011 Cancer screening using “AminoIndex Technology.” Ningen Dock 26(3):454–466.

[ajhb22787-bib-0010] Pedersen EB , Jorgensen ME , Pedersen MB , Siggaard C , Sorensen TB , Mulvad G , Hansen JC , Torstensen AM , Aagaard O , Skjoldborg H. 2006 Plasma amino acids in Greenlanders and Danes: influence of seasons, residence, ethnicity, and diet. Am J Hum Biol 18(1):99–111. 16378345 10.1002/ajhb.20460

[ajhb22787-bib-0011] Rhee EP , Ho JE , Chen MH , Shen D , Cheng S , Larson MG , Ghorbani A , Shi X , Helenius IT , O'Donnell CJ , Souza AL , Deik A , Pierce KA , Bullock K , Walford GA , Vasan RS , Florez JC , Clish C , Yeh JR , Wang TJ , Gerszten RE . 2013 A genome‐wide association study of the human metabolome in a community‐based cohort. Cell Metab 18(1):130–143. 23823483 10.1016/j.cmet.2013.06.013 PMC3973158

[ajhb22787-bib-0012] Shimbo K , Oonuki T , Yahashi A , Hirayama K , Miyano H. 2009a Precolumn derivatization reagents for high‐speed analysis of amines and amino acids in biological fluid using liquid chromatography/electrospray ionization tandem mass spectrometry. Rapid Commun Mass Spectrom 23(10):1483–1492. 19350529 10.1002/rcm.4026

[ajhb22787-bib-0013] Shimbo K , Yahashi A , Hirayama K , Nakazawa M , Miyano H. 2009b Multifunctional and highly sensitive precolumn reagents for amino acids in liquid chromatography/tandem mass spectrometry. Anal Chem 81(13):5172–5179. 19480430 10.1021/ac900470w

[ajhb22787-bib-0014] Yamakado M , Tanaka T , Nagao K , Ishizaka Y , Mitushima T , Tani M , Toda A , Toda E , Okada M , Miyano H , et al. 2012 Plasma amino acid profile is associated with visceral fat accumulation in obese Japanese subjects. Clin Obesity 2(1‐2):29–40. 10.1111/j.1758-8111.2012.00039.x 25586045

[ajhb22787-bib-0015] Yamamoto H , Kondo K , Tanaka T , Muramatsu T , Yoshida H , Imaizumi A , Nagao K , Noguchi Y , Miyano H. 2015 Reference intervals for plasma free amino acid in a Japanese population. Ann Clin Biochem (in press). 10.1177/0004563215583360 25829462

[ajhb22787-bib-0016] Yoshida H , Kondo K , Yamamoto H , Kageyama N , Ozawa S , Shimbo K , Muramatsu T , Imaizumi A , Mizukoshi T , Masuda J , Nakayama D , Hayakawa Y , Watanabe K , Mukaibatake K , Miyano H . 2015 Validation of an analytical method for human plasma free amino acids by high‐performance liquid chromatography ionization mass spectrometry using automated precolumn derivatization. J Chromatogr B Analyt Technol Biomed Life Sci 998–999:88–96. 10.1016/j.jchromb.2015.05.029 26186723

